# The Contagion of Puerperal Fever

**Published:** 1875-07

**Authors:** 


### THE CONTAGION OF PUERPERAL FEVER.

The contagious nature of puerperal fever, and the various ways in which it may be communicated, is a question of the greatest importance to both the profession and the public. A knowledge of what has been written on this point, and of the many interesting cases bearing on it, ought to be in the possession of every one who intends to practice midwifery, for without such knowledge he may unconsciously carry with him the contagion, and communicate it to those under his care; and even with such knowledge, it may be in some instances impossible to avoid becoming the agent of this terrible disease. Puerperal fever is a term which embraces a number of diseases having different causes, symptoms and anatomical lesions. One class of these maladies is that brought on in the puerperal woman by contact with the poison of an acute specific disease, as typhus or erysipelas. In these cases there can be no question of the contagious character of the affection, for instances are recorded in which the puerperal woman has been the means of

*Such an arrangement is, we are informed, figured in Ziemssen's New Cyclopædia of the Practice of Medicine, in the chapter on Diseases of the Nasal Passages.

communicating the original disease to a third person. Another class of cases is that which is due to absorption by the vagina of a poison introduced by the examining finger of the accoucher. The poison in these cases may be the cadaveric or decomposing animal matter, such as the discharge from the generative organs of a patient suffering from puerperal fever; or it may the contagion of an acute specific disease. There is a third class of diseases which is included under the term puerperal fever, which is generated within the body of the patient, the cause being decomposition and absorption of coagula or portions of the placenta retained in the uterus, or the breaking up and entrance into the circulation of a clot which has formed in the uterine vessels. The form of the disease depending on the introduction into the vagina or the formation within the body of a poison other than the acute specific contagion, is not contagious in the sense that typhus fever and erysipelas are, but it is certain that it is not unfrequently communicated by the intervention of a third person, that person being the medical attendant. Numerous instances are on record of outbreaks of puerperal fever, in which all or almost all the cases occurred in the practice of one man. Dr. Armstrong says that out of forty-three cases which occurred in Sunderland in 1813, forty happened in the practice of one surgeon and his assistant. Dr. Gordon observed, with reference to the epidemic which took place in Aberdeen in the years 1789–90, that the disease attacked such women only as were visited or delivered by a practitioner, or taken care of by a man who had previously attended patients affected with the same disorder. Roberton states that from December 3, 1830, to January 4, 1831, a midwife attended thirty patients for a public charity; sixteen of these were attacked with puerperal fever, and they all died. In the same month, 380 women were delivered by other midwives for that institution, but none of the 380 suffered in the smallest degreee. Evidence of this kind might easily be multiplied, but we have said enough to show that the disease is in some cases contagious in the sense typhus is, and that in all cases it is communicable by the accoucher or midwife. And this is not to be wondered at, for we know how difficult—nay, how impossible—it is for some time to remove from the hands which have been engaged in making a post-mortem examination the odor of the cadaver, even by the most abundant ablutions and the careful use of disinfectants. We know, also, what a small quantity of septic poison is sufficient to engender a fatal disease even in a healthy person; but in the puerperal woman the poison acts at a great advantage, because her system is in a more or less exhausted state after the parturient efforts, and it is engaged in throwing off waste products—the wasting from severe muscular action and from the involution going on in the uterus. Besides, the poison, when communicated by the examining finger, is introduced to a part capable of absorbing rapidly, and one which at that time is wounded, bruised and sore from the mechanical violence to which it has been subjected. Under these circumstances, the patient is peculiarly susceptible to the slightest contagion or inoculation. Again, practitioners of midwifery find it their duty frequently, during the first few days after labor, to examine the vaginal discharge. In this manner their hands become more or less tainted; and if the patient be suffering from puerperal fever, this taint is sufficient, even after the most careful washing and use of disinfectants, to communicate the disease to a patient examined soon after. With midwives it is far worse, for generally they act both as nurse and midwife to the poor patients on whom they attend; and thus they have daily to perform the part of nurses, and consequently have their hands, however careful they may be, always tainted with discharge. Add to this the slightest carelessness in the use of soap and water and disinfectants, and there is at once formed a propagator of disease who carries death wherever she goes. This double function of midwives is probably the reason that, in recent outbreaks, they have played a more prominent part than professional men. What, then, are the duties of the medical practitioner in whose practice a case of puerperal fever has occurred? He may change his clothes, he may take any number of baths, he may use all sorts of disinfectants, and still the fatal poison may hang about him, and he may be the bearer of it. Under these circumstances he should refrain from practicing midwifery for a season. It would be wantonly wicked in us were we to speak hesitatingly when such issues are at stake. The danger to the public arising from the non-observance of the precautions above named are incalculable, as we have seen in the outbreak in the neighborhood of the Wandsworth road and at Coventry; and whatever sacrifice the medical man may have to make in carrying out the precautions named, we unhesitatingly say he should make it. In the majority of cases, however, the sacrifice to be made would be very light indeed. Three years ago an outbreak of puerperal fever took place in a provincial town of considerable size. On investigation it was found that all the cases had occurred in the practice of one surgeon and one midwife. The members of the profession located in the town met and requested the gentleman in question to refrain from practicing midwifery for one month, and at the same time agreed to attend his cases for him. The midwife was likewise requested not to attend cases for a similar period. This was agreed to, and no new cases occurred after that date. This illustrates well the line of conduct that should be adopted by the profession in any town where this terrible disease may happen to appear. The unfortunate practitioner in whose practice the case has occurred should bring it before the profession, and should cease from attending midwifery for a time. On the other hand, his professional brethren should help him through his difficulty, and make his loss as small as possible by attending his cases for him during the period of his abstention.—*Lancet.*

